# Cardiovascular Manifestations of Cystic Fibrosis

**DOI:** 10.3390/jcm15207988

**Published:** 2026-10-15

**Authors:** Keshav Garg, Ankit Agrawal, Ahmad Mustafa, Mohab Elnashar, Gunner Gilbert, Akiva Rosenzveig, Arnav Lakkimsetti, Elio Haroun, Aro Daniela Arockiam, Karim Hoyek, Clement Tan, Tom Kai Ming Wang

**Affiliations:** 1 Department of Internal Medicine, University of Arkansas for Medical Sciences, Little Rock, AR 72205, USA; kgarg@uams.edu (K.G.); amustafa@uams.edu (A.M.); melnashar@uams.edu (M.E.); alakkimsetti@uams.edu (A.L.); 2 Department of Cardiovascular Medicine, University of Arkansas for Medical Sciences, Little Rock, AR 72205, USA; aagrawal@uams.edu; 3 Department of Internal Medicine, University of Arkansas for Medical Sciences, Fayetteville, AR 72703, USA; grgilbert@uams.edu; 4 Department of Cardiovascular Medicine, Heart, Vascular and Thoracic Institute, Cleveland Clinic, Cleveland, OH 44195, USA; rosenza@ccf.org (A.R.); hoyeckk2@ccf.org (K.H.); tanc7@ccf.org (C.T.); 5 Department of Internal Medicine, Yale New-Haven Health System, New Haven, CT 06510, USA; elio.haroun@hotmail.com; 6 Department of Internal Medicine, Unity Health, Searcy, AR 72143, USA; daniearo25@gmail.com; 7 Green Lane Cardiovascular Service, Te Toka Tumai Auckland City Hospital, Auckland 1023, New Zealand

**Keywords:** cystic fibrosis, CFTR, cardiovascular disease, cardiomyopathy, pulmonary hypertension, CFTR modulators, myocardial fibrosis, cardiac MRI

## Abstract

Cystic fibrosis (CF) has undergone a remarkable transformation from a fatal childhood disease to a chronic adult condition, with a median predicted survival now exceeding 50 years following the introduction of highly effective CFTR modulator therapy (HEMT). This demographic shift has unmasked cardiovascular complications that were previously unrecognized due to premature mortality. In a multinational propensity-matched cohort, CF conferred a 65% higher risk of major adverse cardiac events (MACEs), including myocardial infarction, heart failure, and atrial fibrillation, versus matched controls (Frost et al., HR 1.65). In a separate Canadian population-based cohort, cardiovascular disease occurred at 2.9 times the rate of the general population and at least 20 years earlier (Arya et al., RR 2.9). Cardiac involvement extends beyond the traditional paradigm of cor pulmonale. CFTR is expressed in cardiomyocytes, vascular endothelium, and smooth muscle, where its dysfunction disrupts ion homeostasis, impairs nitric oxide-dependent vasodilation, and promotes oxidative stress. Clinically, these mechanisms manifest as biventricular dysfunction detectable by strain echocardiography and cardiac MRI even when ejection fraction is preserved, diffuse myocardial fibrosis, endothelial dysfunction and accelerated arterial stiffness from childhood, increased arrhythmia susceptibility, and, in a single-center cohort, an unexpectedly high prevalence of pericardial calcification (18%) on chest CT that awaits independent replication. Paradoxically, while HEMT has dramatically improved pulmonary outcomes, it introduces new cardiovascular considerations including hypertension, dyslipidemia, and obesity, and cross-sectional data in modulator-treated cohorts show persistent cardiac abnormalities, though whether these predate treatment or would differ without it is unknown. This review synthesizes the genetic, molecular, and clinical dimensions of cardiovascular disease in CF; distinguishes direct CFTR-mediated cardiac effects from indirect consequences of chronic lung disease; examines vascular, electrophysiological, valvular, and pericardial manifestations; and discusses management implications including CFTR modulator therapy, exercise, nutritional paradigm shifts, and transplantation considerations.

## 1. Introduction

Cystic fibrosis (CF) is an autosomal recessive genetic disease that results from mutations in the cystic fibrosis transmembrane conductance regulator (CFTR) gene, which encodes a protein involved in transport and regulation of chloride and sodium ions through epithelial cell membranes throughout the body [1,2]. The loss of function of CFTR leads to thick secretions obstructing the exocrine glands, predisposing to chronic respiratory infections, pancreatic insufficiency and progressive multiorgan dysfunction [1,2]. CF predominantly affects Caucasian populations, with an incidence of approximately 1 in 3000–3500 live births in the United States, where roughly 31,450 individuals are currently diagnosed [1,2,3]. The F508del mutation is present in over 85.5% of US patients [1,2].

The trajectory of CF has changed fundamentally. Median predicted survival has risen from 17 years in 1970 to 53.1 years for individuals born during 2017–2021, now exceeding 50 years in Canada, Australia, New Zealand, European countries, and the United States [1,2,4]. This transformation has been driven primarily by CFTR modulators, small molecules that correct the underlying molecular defect. The 2019 approval of elexacaftor/tezacaftor/ivacaftor (ETI), which is eligible for approximately 90% of the US CF population, improved FEV_1_ by 13.8 percentage points (95% CI, 12.1–15.4%) and reduced pulmonary exacerbations by 63% (rate ratio 0.37; 95% CI, 0.25–0.55) [1]. Alongside highly effective modulator therapy (HEMT), advances in newborn screening, antimicrobial therapy, mucolytics, nutritional support, and lung transplantation have contributed to this transformation [1]. Throughout this review, HEMT denotes the class of highly effective modulator therapies (including ETI and next-generation combinations such as vanzacaftor/tezacaftor/deutivacaftor), whereas ETI refers specifically to elexacaftor/tezacaftor/ivacaftor. In most countries with established CF care, adults now outnumber children, and the aging population is encountering complications that were previously unrecognized [2].

Cardiovascular disease is chief among these emerging complications. Historically, cardiac involvement in CF was understood exclusively through the lens of cor pulmonale, progressive right heart failure secondary to irreversible lung disease [5]. Patients rarely survived long enough for other cardiovascular manifestations to become clinically apparent [6,7]. However, converging lines of evidence now demonstrate that cardiac disease in CF arises from multiple mechanisms: direct CFTR dysfunction in vascular endothelium and smooth muscle, chronic systemic inflammation from persistent lung infection, CF-related diabetes, chronic hypoxia, and, increasingly, traditional risk factors as patients age on HEMT [6,7].

Recent population-based studies have quantified this burden. A Canadian cohort demonstrated that individuals with CF develop cardiovascular disease at 2.9 times the rate of the general population (RR 2.9; 95% CI 2.6–3.4), with events occurring at least 20 years earlier [8]. A multinational propensity-matched analysis of over 11,000 CF patients confirmed a 65% higher risk of MACEs, myocardial infarction, heart failure, and atrial fibrillation, compared with matched controls (HR 1.65; 95% CI 1.40–1.95), a risk equivalent to or exceeding that of systemic lupus erythematosus, rheumatoid arthritis, and HIV [9]. Cardiac admissions among CF patients in the United States increased by 16.4% per year from 2016 to 2022, with higher in-hospital mortality for cardiac versus non-cardiac admissions [10]. Despite this evidence, the CF Foundation care model does not currently include systematic cardiac assessment beyond blood pressure monitoring [11].

This review aims to synthesize the current evidence on cardiovascular manifestations in CF, distinguish direct CFTR-mediated cardiac effects from indirect consequences of pulmonary disease, address clinical implications for screening and management, and identify critical research gaps as the CF population ages into the fifth, sixth, and seventh decades of life.

### Review Methodology

This is a narrative review. PubMed/MEDLINE, Embase, and the Cochrane Library were searched from inception to May 2026 using combinations of the terms “cystic fibrosis,” “CFTR,” and cardiovascular terms (“cardiomyopathy”, “myocardial fibrosis”, “pulmonary hypertension”, “right/left ventricular dysfunction”, “strain echocardiography”, “cardiac MRI”, “arterial stiffness”, “endothelial dysfunction”, “arrhythmia”, “atrial fibrillation”, “pericardial”, “MACE”, “CFTR modulator”). English-language original studies, systematic reviews, society guidelines, and United States Food and Drug Administration (FDA) labels were eligible; reference lists of key articles were hand-searched. Given the heterogeneous evidence base, ranging from mechanistic knockout/overexpression models and autopsy series to small single-center imaging cohorts, retrospective databases, and multinational cohorts, studies were weighted by design, sample size, and replication across independent cohorts, and mechanistic (animal/in vitro) evidence is distinguished from human clinical evidence throughout. Design and sample size for the pivotal evidentiary studies are summarized in Supplementary Table S1 to allow readers to calibrate confidence; small single-center imaging studies are interpreted with caution given the potential for publication bias favoring positive findings. During the preparation of this work, the authors used Google Gemini (Mountain View, California, United States) to generate figure graphics based on author-developed conceptual designs and content. The authors reviewed and edited all output and take full responsibility for the content of the publication.

## 2. Pathophysiology of CF Cardiovascular Manifestations

### 2.1. Continuum and Pathway Model of CF-Mediated Cardiovascular Disease

Cardiovascular disease in CF arises from the convergence of four interrelated pathways: (1) direct CFTR dysfunction in cardiomyocytes, vascular endothelium, and smooth muscle, which disrupts ion homeostasis, impairs nitric oxide-dependent vasodilation, and alters contractile physiology [7,12]; (2) pulmonary disease-mediated cardiac remodeling, whereby progressive airway obstruction, chronic hypoxia, and pulmonary vascular remodeling impose chronic pressure and volume overload on the right ventricle (RV), with secondary effects on the left ventricle (LV) through ventricular interdependence [13,14]; (3) systemic inflammation and metabolic dysfunction, including chronic pro-inflammatory cytokine elevation, CF-related diabetes, and nutritional derangements, which promote endothelial dysfunction, oxidative stress, and accelerated vascular aging [6,9]; and (4) emerging conventional cardiovascular risk factors in the post-HEMT era, including hypertension from corrected salt-wasting, dyslipidemia, obesity, and insulin resistance, which compound residual CF-specific cardiac vulnerability as patients achieve improved CFTR function and extended survival [8,15,16].

These four pathways operate across a continuum whose dominant drivers have shifted between two distinct eras. In the pre-HEMT era, pathways 2 and 3 dominated the clinical picture: progressive pulmonary disease led to chronic hypoxemia, pulmonary hypertension, and RV dysfunction culminating in cor pulmonale, while malnutrition and chronic infection amplified myocardial and vascular injury. Patients who survived to lung transplantation faced the cardiovascular consequences of chronic immunosuppression, with macrovascular disease prevalence reaching 15.9% by 15 years post-transplant [17,18]. In the post-HEMT era, effective CFTR modulation has substantially attenuated pulmonary vascular remodeling and RV afterload, and improved nutritional status has largely eliminated the malnutrition-driven component of pathway 3. However, pathway 1, direct CFTR effects on cardiomyocytes, persists, as suggested by a single cross-sectional cohort (Duus et al., n = 104, 93% on modulators) in which 44% met a composite definition of abnormal cardiac function, based on predefined thresholds for LV diastolic function, LV systolic function (global longitudinal strain), or RV systolic function, with any one abnormal parameter classifying a participant as positive, compared with matched controls [19]. Because this is a single-center, cross-sectional study, the figure awaits independent replication and cannot establish causation or temporality. Simultaneously, pathway 4 has emerged as a new and increasingly important contributor: correction of salt-wasting leads to hypertension, weight gain promotes obesity and dyslipidemia, and the aging CF population encounters traditional atherosclerotic risk factors that were previously irrelevant in a population with limited life expectancy [8,9,15,16,20].

**Figure 1:**
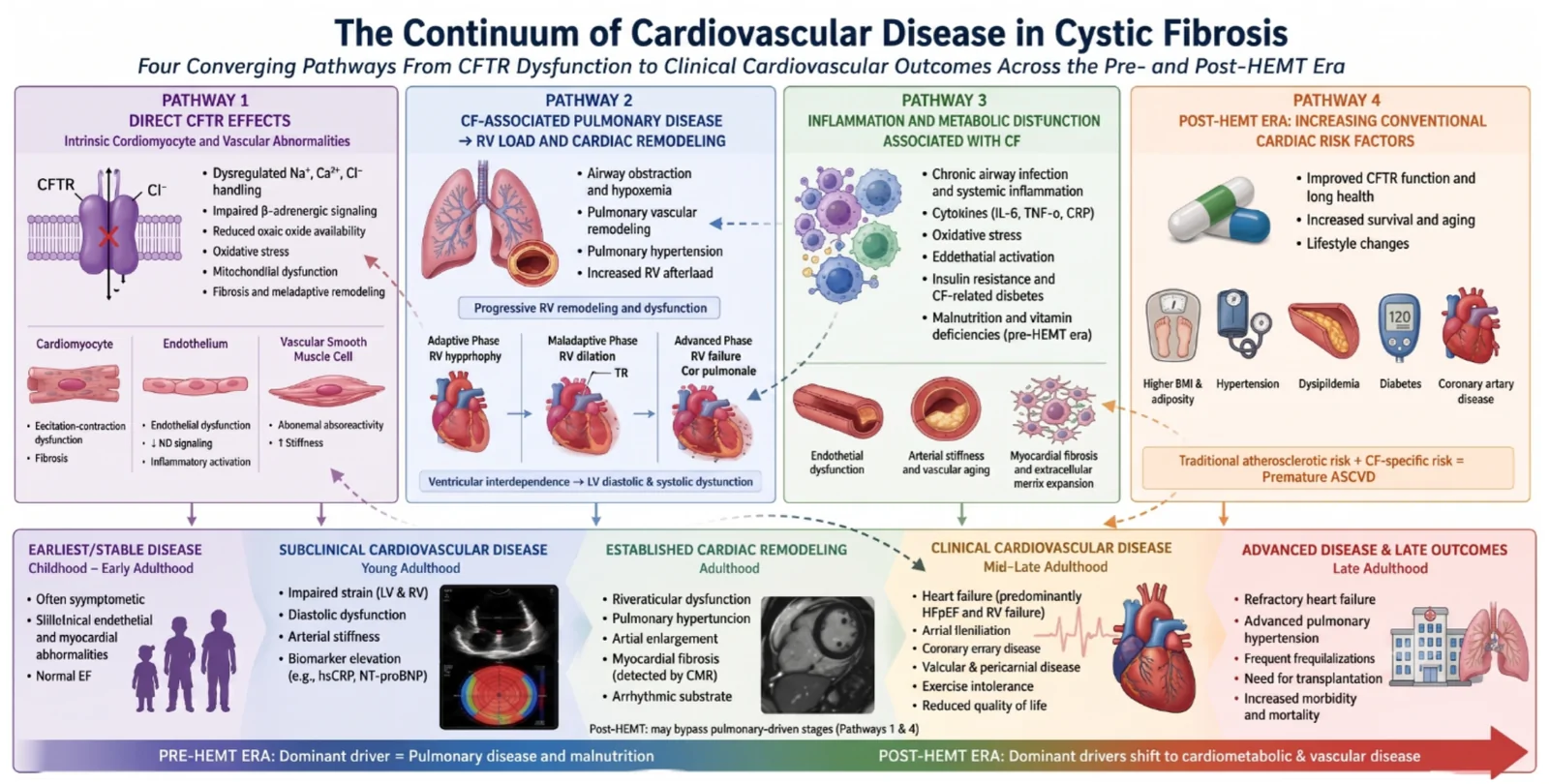
The Continuum of Cardiovascular Disease in Cystic Fibrosis. The upper panel depicts four converging pathways: (1) direct CFTR-mediated effects on cardiomyocytes, endothelium, and vascular smooth muscle; (2) pulmonary disease-driven RV remodeling; (3) systemic inflammation and metabolic dysfunction; and (4) emerging conventional risk factors in the post-HEMT era. The lower panel illustrates the clinical continuum across five stages from childhood through late adulthood, with dominant drivers shifting from pulmonary disease (pre-HEMT) to cardiometabolic and vascular disease (post-HEMT). Abbreviations: ASCVD, atherosclerotic cardiovascular disease; BMI, body mass index; Ca^2+^, calcium; CFTR, cystic fibrosis transmembrane conductance regulator; Cl^−^, chloride; CMR, cardiac magnetic resonance; CRP, C-reactive protein; EF, ejection fraction; HEMT, highly effective modulator therapy; HFpEF, heart failure with preserved ejection fraction; HFrEF, heart failure with reduced ejection fraction; hsCRP, high-sensitivity C-reactive protein; IL-6, interleukin-6; LV, left ventricular; Na^+^, sodium; NO, nitric oxide; NT-proBNP, N-terminal pro-B-type natriuretic peptide; RV, right ventricular; TNF-α, tumor necrosis factor-alpha; TR, tricuspid regurgitation.

The clinical continuum can be conceptualized across five stages (Figure 1, bottom panel). In childhood and early adulthood, subclinical endothelial and myocardial abnormalities are detectable by advanced imaging despite normal ejection fraction and often normal lung function. In young adulthood, impaired biventricular strain, diastolic dysfunction, arterial stiffness, and biomarker elevation become apparent. By adulthood, established cardiac remodeling—including biventricular dysfunction, pulmonary hypertension, atrial enlargement, and myocardial fibrosis—is present. In mid-to-late adulthood, clinical cardiovascular disease manifests as heart failure [predominantly heart failure with preserved ejection fraction (HFpEF)], atrial fibrillation, coronary artery disease, and valvular and pericardial disease. In advanced disease, refractory heart failure, advanced pulmonary hypertension, and the need for transplantation define the terminal cardiovascular phenotype. Importantly, in the post-HEMT era, patients may not follow this linear trajectory—those with well-controlled pulmonary disease may bypass the pulmonary-driven stages and develop cardiovascular disease primarily through pathways 1 and 4, entering the continuum at different stages depending on their metabolic and cardiac risk profile.

This framework underscores that while the dominant cardiovascular phenotype has shifted from cor pulmonale to a mixed picture of subclinical cardiomyopathy and emerging atherosclerotic disease, the total cardiovascular burden in CF is not decreasing, it is transforming.

### 2.2. Genetic and Molecular Basis of Cardiac Involvement

#### 2.2.1. CFTR Expression in the Heart and Vasculature

The CFTR protein is a cyclic adenosine monophosphate (cAMP)-regulated chloride and bicarbonate channel classically associated with epithelial ion transport. Although cardiac expression is lower than in epithelial organs, CFTR is detectable in cardiomyocytes, vascular endothelial cells, and vascular smooth muscle cells [21,22].

In the vascular endothelium, CFTR regulates nitric oxide bioavailability and endothelial-dependent vasodilation; loss of function is associated with impaired endothelial signaling, increased oxidative stress, and reduced vasodilatory capacity [23]. In vascular smooth muscle, CFTR influences vascular tone through effects on chloride flux and membrane polarization [24]. These findings support a role for CFTR in maintaining vascular homeostasis and suggest that its dysfunction may contribute to increased vascular stiffness and altered hemodynamics observed in individuals with CF. Within cardiomyocytes, CFTR contributes to intracellular ion homeostasis through interactions with chloride currents that influence membrane potential and excitation–contraction coupling, and may modulate calcium handling indirectly, thereby affecting contractile function and myocardial relaxation [25,26].

#### 2.2.2. Altered Ion Handling and Excitation–Contraction Coupling

Disruption of CFTR function alters intracellular chloride gradients, which indirectly influence calcium cycling through effects on membrane depolarization and calcium channel activity [23,27]. In CFTR-knockout cardiomyocytes, Sellers et al. demonstrated compensatory activation of Calcium/calmodulin-dependent kinase II (CaMKII) and calcium-activated chloride channels (CaCCs) to maintain normal contraction rates, a maladaptive dependency that may predispose to arrhythmia and remodeling [28]. CFTR may also modulate β-adrenergic signaling; impaired function has been associated with blunted cAMP-mediated signaling, potentially reducing the efficiency of calcium reuptake into the sarcoplasmic reticulum and contributing to delayed relaxation [27,28]. In cardiac-specific CFTR-knockout mice, Jiang et al. found increased myocardial twist and torsion at baseline, independent of pulmonary disease, along with decreased aortic diameter, indicating a hypercontractile baseline state that may trigger adverse remodeling [27].

#### 2.2.3. Endothelial Dysfunction and Vascular Signaling

Loss of CFTR function in the vascular endothelium reduces NO production, increases reactive oxygen species generation, and impairs endothelial signaling, promoting a vasoconstrictive and pro-inflammatory phenotype [24,25,26]. Totani et al. demonstrated that CFTR blockade in human endothelial cells suppresses insulin-stimulated endothelial nitric oxide synthase phosphorylation and NO generation, increases endothelial permeability, disrupts VE-cadherin junctions, and triggers microvesicle release. These changes promote a vasoconstrictive and pro-inflammatory vascular phenotype [29]. Transcriptomic analysis by Declercq et al. confirmed downregulation of proliferation, migration, and autophagy pathways alongside persistent endothelial activation with increased leukocyte adhesion in CFTR-silenced endothelial cells, findings validated in CFTR-knockout mice and partially rescued by CFTR modulators VX-770 and VX-809 [25]. In vascular smooth muscle, CFTR deficiency aggravates angiotensin II-induced vasoconstriction through dysregulated calcium influx and the RhoA/ROCK pathway [30]. Collectively, these in vitro and animal data support a mechanistic role for the endothelium as an active contributor to the CF vascular phenotype; whether this translates into clinically significant vascular disease in humans remains to be established.

#### 2.2.4. Arrhythmogenesis

Transgenic overexpression of human CFTR in the mouse heart leads to stress-related sudden death. In vivo electrophysiology demonstrates that, following isoproterenol, CFTR-overexpressing mice develop marked conduction slowing, high-grade AV block, and easily inducible ventricular tachycardia, establishing that dysregulated cardiac chloride channel activity is directly arrhythmogenic [31]. Together with the loss-of-function data from Sellers et al., these gain- and loss-of-function models establish that precise CFTR dosage is critical for electrical stability [28].

#### 2.2.5. Fibrotic Remodeling

Chronic cellular stress from altered ion handling and oxidative injury activates cardiac fibroblasts and promotes extracellular matrix deposition. Cardiac MRI in adults with stable CF demonstrates LV hypertrophy driven predominantly by extracellular matrix expansion; extracellular volume (ECV) was significantly elevated (27.6% vs. 24.8%; *p* = 0.030), and extracellular matrix mass increased (27.5 ± 3.4 vs. 23.6 ± 5.2 g; *p* = 0.006) compared with controls [13]. During acute exacerbations, LVEF declined acutely with evidence of myocardial edema that correlated strongly with airflow limitation severity (r = −0.726; *p* = 0.017) [13]. These findings demonstrate that LV hypertrophy in CF is driven by diffuse interstitial fibrosis, a substrate for both diastolic dysfunction and arrhythmogenesis, and that the CF myocardium has diminished reserve and is vulnerable to inflammatory insults.

#### 2.2.6. Genotype–Phenotype Correlations

CFTR mutations are categorized into functional classes based on their effects on protein production, processing, and channel activity, with classes I–III generally associated with more severe loss of function and classes IV–VI with residual CFTR activity [1,32]. The most common mutation, F508del, results in defective protein folding and markedly reduced cell-surface CFTR expression. Zebrak et al. characterized 18 cases of infantile myocardial fibrosis and necrosis presenting as sudden cardiac death before age 2, predominantly in F508del homozygotes, with a consistent phenotype of profound ECG changes, mild pulmonary involvement, and severe pancreatic insufficiency—implicating a direct myocardial effect of severe CFTR dysfunction compounded by nutritional deficiency [33]. Familial concordance in affected siblings further supported a genetic basis [33].

Individuals with more severe CFTR dysfunction may be at higher risk for systemic complications that indirectly impact the cardiovascular system through earlier onset and greater progression of pulmonary disease, increasing the likelihood of chronic hypoxemia, pulmonary vascular remodeling, and right ventricular strain [19]. Variability in clinical expression even among individuals with identical mutations, driven by modifier genes, environmental exposures, and differences in inflammatory burden, complicates attempts to establish clear genotype–phenotype relationships for cardiovascular outcomes.

### 2.3. Pulmonary–Cardiac Coupling

The RV is a thin-walled, flow-generating chamber highly sensitive to afterload. In CF, progressive airway obstruction and parenchymal destruction elevate pulmonary vascular resistance (PVR) through chronic hypoxic pulmonary vasoconstriction (HPV), inflammation-driven vascular remodeling, and mechanical compression from hyperinflated lungs [34,35]. At the molecular level, CFTR participates directly in HPV signaling; hypoxia induces CFTR to interact with TRPC6 channels in pulmonary artery smooth muscle cells, facilitating calcium-mediated vasoconstriction via sphingolipid pathways [36]. CFTR deficiency therefore impairs adaptive HPV while simultaneously promoting maladaptive remodeling, worsening ventilation–perfusion mismatch [36,37].

Recent micro-CT analysis of explanted CF lungs has challenged the view that pulmonary vascular disease is purely secondary to airway obstruction. Vermaut et al. demonstrated distal arteriole narrowing and fibrotic wall changes even in regions where terminal bronchioles remained patent, suggesting an intrinsic CFTR-related vasculopathy [38]. This inference derives from explanted end-stage lungs and cannot exclude the contribution of chronic infection and remodeling; it is hypothesis-generating rather than confirmatory. This is corroborated by findings that CFTR expression is reduced in pulmonary arteries of patients with idiopathic pulmonary arterial hypertension, and that pharmacological CFTR inhibition in animal models produces pulmonary hypertension through exaggerated vascular cell proliferation [39]. As PVR rises, the RV initially compensates through concentric hypertrophy; when this adaptive response is exhausted, the ventricle dilates, functional tricuspid regurgitation develops, and overt cor pulmonale ensues [40,41]. The clinical spectrum, prevalence, and prognostic markers of RV disease are discussed in Section 3.

### 2.4. LV Involvement

#### 2.4.1. Ventricular Interdependence

The traditional view of cardiac disease in CF has centered on the RV. However, the two ventricles share the interventricular septum, pericardial constraint, and coronary perfusion, a relationship termed ventricular interdependence. Jacobstein et al. demonstrated that in CF patients with severe cor pulmonale, massive RV dilation flattened and compressed the LV along its minor dimension, producing dyskinetic septal motion and impaired LV filling [42]. Allen et al. extended these observations across the disease spectrum, showing that LV cavity dimensions were smaller than normal in both systole and diastole even in patients with mild disease, while septal and posterior wall thickness increased with disease severity [43]. These mechanical effects represent one pathway to LV dysfunction in CF—but accumulating evidence suggests it is not the only one.

#### 2.4.2. Evidence for a Primary CFTR-Related Cardiomyopathy

The concept of a primary, CFTR-related cardiomyopathy, distinct from the secondary effects of lung disease, has gained traction from converging experimental and clinical evidence, though it should be noted that this entity has been characterized primarily through small imaging studies, experimental models, and autopsy series rather than large population-based investigations [12,14]. The molecular basis for this entity, including compensatory CaMKII/CaCC activation in CFTR-knockout cardiomyocytes and the hypercontractile baseline state in cardiac-specific CFTR-knockout mice, is discussed in the Genetic and Molecular Basis Section [27,28].

Clinically, the strongest argument comes from studies demonstrating LV dysfunction that does not correlate with pulmonary function. Beyond the Sellers et al. findings in adults, Eising et al. studied children aged 3–12 years with CF who had normal lung function and no differences in anthropometrics or blood pressure compared with controls, yet demonstrated significantly reduced global LV and RV strain after adjustment for lung function [44,45]. These findings in young children with preserved pulmonary function are difficult to attribute to overt secondary hemodynamic effects; however, as a cross-sectional cohort adjusted only for lung function, the study cannot exclude unmeasured confounders such as subclinical systemic inflammation, occult hypoxemia not captured by spirometry, or nutritional status, and is therefore hypothesis-generating rather than confirmatory of an intrinsic myocardial process.

The pathological basis was first described in autopsy series by Zebrak et al., who identified myocardial fibrosis and necrosis in 18 CF infants presenting as sudden cardiac death before age 2, predominantly in F508del homozygotes with profound ECG changes, mild pulmonary involvement, and severe pancreatic insufficiency [33]. Cardiac MRI has provided the most direct in vivo corroboration: Lagan et al. demonstrated that stable CF is associated with LV hypertrophy driven by extracellular matrix expansion rather than myocyte hypertrophy, with significantly elevated ECV and extracellular matrix mass, while acute exacerbations produced myocardial edema whose severity correlated with airflow limitation [13]. The clinical echocardiographic and hemodynamic evidence for LV and biventricular dysfunction is presented in Section 3.

### 2.5. Metabolic Dysfunction in CF Cardiovascular Disease

#### 2.5.1. Indirect Systemic Drivers

Cardiac involvement in CF is not solely a consequence of intrinsic CFTR dysfunction but also reflects the cumulative impact of chronic systemic stressors. Among these, persistent hypoxia and inflammation form a central, interrelated axis that contributes to both vascular and myocardial dysfunction.

#### 2.5.2. Chronic Hypoxia

Progressive pulmonary disease in CF leads to ventilation–perfusion mismatch and chronic hypoxemia, particularly in advanced stages. Sustained hypoxia promotes pulmonary vasoconstriction and structural remodeling of the pulmonary vasculature through activation of hypoxia-inducible factors (HIFs), which regulate gene expression programs involved in angiogenesis, metabolism, and vascular tone [46]. While initially adaptive, prolonged HIF activation contributes to maladaptive vascular remodeling and increased pulmonary vascular stiffness. The resulting chronic pressure overload on the RV drives compensatory hypertrophy and, over time, RV dilation and dysfunction—the hemodynamic cascade detailed in the RV Dysfunction Section [47]. In parallel, hypoxia may impair myocardial energetics by limiting oxygen availability for oxidative phosphorylation, exacerbating underlying mitochondrial dysfunction and reducing contractile efficiency [46].

#### 2.5.3. Systemic Inflammation and Myocardial Injury

CF is characterized by a chronic pro-inflammatory state driven by recurrent infection and persistent immune activation. Elevated circulating levels of IL-6 and TNF-α have been associated with endothelial dysfunction, oxidative stress, and adverse myocardial remodeling [9]. These inflammatory mediators can directly affect cardiomyocyte function by altering calcium handling, impairing contractility, and promoting apoptotic signaling pathways. The clinical evidence linking inflammatory biomarkers to endothelial injury and vascular dysfunction is detailed in the Vascular and Endothelial Manifestations Section [48,49,50].

Inflammation also accelerates atherosclerotic processes and microvascular dysfunction, creating a cardiovascular risk profile analogous to other systemic inflammatory conditions. As discussed in the Introduction, the Frost et al. multinational analysis confirmed that both traditional risk factors and CF-specific inflammatory burden (indexed by intravenous antibiotic days) independently predict MACEs [9].

#### 2.5.4. Metabolic Contributors

Additional systemic factors further modulate the hypoxia–inflammation axis. Malnutrition and deficiencies in fat-soluble vitamins may impair myocardial function and antioxidant capacity, increasing susceptibility to oxidative injury. CF-related diabetes (CFRD) contributes to microvascular dysfunction and may potentiate myocardial injury through mechanisms similar to those observed in diabetic cardiomyopathy [7]. These comorbidities often coexist with advanced pulmonary disease, amplifying the overall cardiovascular burden.

The metabolic landscape is further complicated by the HEMT era. Correction of the salt-wasting phenotype, weight gain, and dyslipidemia introduce new cardiometabolic risk factors that compound the residual CF-specific cardiac vulnerability, considerations discussed in detail in the Management Section [15,51].

### 2.6. Vascular and Endothelial Dysfunction

#### 2.6.1. Endothelial Dysfunction

Endothelial dysfunction, defined as impaired nitric oxide (NO)-dependent vasodilation, is an early and consistent vascular abnormality in CF. Poore et al. provided the first clinical evidence, demonstrating that brachial artery flow-mediated dilation (FMD) was significantly reduced in young, otherwise healthy CF patients compared with matched controls (4.9 ± 2.6% vs. 7.5 ± 3.1%; *p* = 0.018), with FMD correlating with both pulmonary function and exercise capacity [52]. Subsequent work confirmed that both conduit artery and microvascular endothelial dysfunction are present in CF [53].

The molecular mechanisms underlying endothelial dysfunction—including CFTR-mediated regulation of NO bioavailability, reactive oxygen species generation, endothelial barrier integrity, and the pro-inflammatory transcriptomic signature of CFTR-deficient endothelial cells—are detailed in the Genetic and Molecular Basis Section [23,29]. Of particular clinical relevance, Tucker et al. demonstrated that a single dose of an antioxidant cocktail significantly improved FMD in CF patients compared with placebo, providing direct evidence that oxidative stress, rather than structural vascular damage alone, drives endothelial dysfunction in this population and suggesting a potentially modifiable therapeutic target [53].

In the microvascular compartment, Kreslová et al. demonstrated age-related progression of microvascular dysfunction, with biomarkers of endothelial activation—E-selectin, VCAM-1, and high-sensitivity CRP—significantly elevated from childhood, preceding the functional decline in reactive hyperemia index (RHI) that becomes apparent in adulthood [54]. This temporal sequence, biomarker elevation preceding functional impairment, mirrors the natural history of endothelial dysfunction in other inflammatory conditions and suggests a window for early intervention.

#### 2.6.2. Arterial Stiffness and Vascular Aging

Arterial stiffness, an independent predictor of cardiovascular events in the general population, is increased in CF from childhood onward. Arterial stiffness is detectable from childhood in CF, with elevated pulse wave velocity and augmentation index documented in pediatric cohorts, as detailed in the Special Populations Section [45,55].

In adults, Pessoa et al. found markedly elevated AIx@75 in CF patients compared with age- and sex-matched controls (29.9 ± 9.4% vs. 16.5 ± 7.2%; *p* < 0.0001), alongside lower stroke volume and cardiac output—a hemodynamic profile consistent with increased LV afterload from stiffened arteries [56].

#### 2.6.3. Pre-Clinical Atherosclerosis: Two Distinct Phenotypes

The relationship between arterial stiffness and preclinical atherosclerosis varies by pancreatic status. Nowak et al. identified two distinct phenotypes: pancreatic-insufficient (PI) patients had increased arterial stiffness (SI: 7.3 ± 1.8 vs. 6.0 ± 1.2 m/s; *p* = 7.1 × 10^−5^) but normal carotid intima–media thickness (IMT), while pancreatic-sufficient (PS) patients had increased IMT (552 ± 69 vs. 456 ± 95 µm; *p* = 0.001) but normal stiffness [57]. SI was independently associated with age, PI status, and Pseudomonas aeruginosa colonization, while PS was the sole independent correlate of IMT [57]. These divergent presentations suggest that the pathways to vascular disease differ by CF phenotype—functional stiffening driven by inflammation and infection in PI patients versus structural intimal thickening in PS patients whose higher-fat diets and better nutritional status may promote traditional atherogenesis.

#### 2.6.4. Chronic Inflammation and Cardiovascular Risk

The chronic inflammatory milieu of CF creates a vascular environment analogous to other systemic inflammatory diseases with established cardiovascular risk. Multiple studies have documented significantly elevated circulating adhesion molecules (sICAM-1, sE-selectin, sVCAM-1), von Willebrand factor, tissue-plasminogen activator, P-selectin, TNF-α, and IL-6 in CF patients, with endothelial injury markers correlating directly with inflammatory cytokines and inversely with lung function [48,49,50]. These adhesion molecules mediate leukocyte recruitment to activated endothelium, a critical initiating event in atherosclerotic plaque formation [58]. Notably, Nowak et al. found that sVCAM-1 elevation in CF did not associate with conventional CF disease characteristics in multivariable analysis, raising the possibility that endothelial dysfunction may partly represent a primary CFTR-related microangiopathy independent of systemic inflammation [50].

In vascular smooth muscle, CFTR deficiency has direct pro-atherogenic consequences beyond the vasoconstrictor effects described in the Molecular Basis Section. Lu et al. showed that CFTR suppresses neointimal formation after vascular injury by inhibiting PDGF-BB-stimulated VSMC proliferation and migration via the JNK/p38/ERK pathway, and that CFTR expression declines in injured arteries, creating a feed-forward cycle of vascular remodeling [59].

The epidemiological data corroborate these mechanistic findings (elevated MACE risk relative to SLE, RA, and HIV; Section 1) [9].

These vascular and endothelial pathways contribute to the clinical manifestations of coronary artery disease and heart failure discussed in Section 3, and inform the diagnostic strategies in Section 5.

## 3. Manifestations of CF Cardiovascular Disease

### 3.1. Right Ventricular Dysfunction & Cor Pulmonale

The pathophysiological basis for RV dysfunction, including CFTR-mediated hypoxic pulmonary vasoconstriction signaling, intrinsic pulmonary vasculopathy, and the adaptive-to-maladaptive remodeling cascade, is detailed in Section 2.3.

#### 3.1.1. The Spectrum of RV Disease

Pulmonary hypertension (PH) affects 36–60% of CF patients undergoing transplant evaluation. In a cohort of 1273 CF transplant recipients, Chandel et al. reported a PH prevalence of 60%, significantly exceeding that in non-CF bronchiectasis (46%; *p* < 0.001). In pediatric PH, prevalence is discussed in the Special Populations Section [60].

RV dysfunction begins well before clinical cor pulmonale. Tissue Doppler studies demonstrate reduced RV systolic velocities and increased wall thickness in clinically stable CF patients, with RV systolic function correlating inversely with CRP and diastolic function tracking with IL-6 levels [61]. Speckle-tracking echocardiography confirms impaired RV strain (−22.0% vs. −29.5% in controls; *p* < 0.001) that correlates with FEV_1_ [62]. Even in the HEMT era, Duus et al. found abnormal cardiac function in 44% of CF patients (defined and discussed in Section 2.1), with male sex and decreasing FEV_1_/FVC being independently predictive [19].

Exercise hemodynamics reveal an additional burden: Hayes et al. showed that mean pulmonary artery pressure rose from 27.8 to 47.2 mmHg during exercise in CF patients referred for transplantation, with PVR increasing fourfold despite unremarkable resting echocardiograms—underscoring the insensitivity of resting studies for detecting hemodynamically significant disease [63]. Classic autopsy data from Ryland and Reid established the histopathological substrate, demonstrating RV hypertrophy in five of six CF patients dying after age 3, accompanied by reduced arterial density, medial muscle thickening, and subintimal fibrosis [64].

#### 3.1.2. Clinical Indicators and Prognostic Markers

Hypoxemia is the strongest predictor of PH in CF. Fraser et al. demonstrated that awake SaO_2_ predicted pulmonary artery systolic pressure (PASP) more accurately than FEV_1_ (*p* = 0.01), with exercise desaturation showing the tightest correlation (r = −0.75; *p* < 0.0001) [46]. Mortality was significantly higher in those with subclinical PH (PASP > 35 mmHg), even without clinical cor pulmonale [46].

Among imaging biomarkers, the pulmonary artery-to-aorta (PA:A) ratio on chest CT has emerged as a practical screening tool. A PA:A ratio >1 independently predicts both PH on catheterization (adjusted OR 4.50) and death or transplant (adjusted HR 2.69) [65]. PA enlargement is present in roughly half of adult CF patients with moderate obstruction and independently predicts future exacerbations [66]. NT-proBNP has limited sensitivity in CF, as population-level values do not differ from controls, though approximately 25% show mild elevations that may signal subclinical cardiac stress [67].

Serial echocardiographic parameters—TAPSE, RV strain, and the TAPSE:PASP ratio as a noninvasive surrogate for RV–PA coupling—offer the most practical approach for longitudinal surveillance [40,41,62]. The prognostic landscape is nuanced: in the largest transplant cohort analysis, PH was paradoxically associated with lower early post-transplant mortality but worse outcomes beyond 3 years, highlighting the need for continued cardiovascular surveillance even after successful transplantation [60].

### 3.2. Left Ventricular and Biventricular Involvement

The mechanistic basis for LV involvement, including ventricular interdependence, experimental evidence for a primary CFTR-related cardiomyopathy, and the pathological substrate of myocardial fibrosis, is detailed in Section 2.4.

#### 3.2.1. LV Systolic and Diastolic Dysfunction

Conventional LVEF has generally been preserved in CF cohorts, which historically led to the conclusion that the left heart is spared [14,68]. Advanced imaging has challenged this dogma. Labombarda et al. demonstrated that despite preserved LVEF, CF patients had significantly reduced LV longitudinal strain of both septal and lateral walls, along with decreased mitral annular systolic and diastolic velocities on tissue Doppler imaging [69]. Sellers et al. confirmed these findings, reporting that strain rate echocardiography identified LV systolic abnormalities in 100% of CF adults studied, with 50% of men and 100% of women showing decreased systolic strain—none detected by conventional echocardiography [44]. This apparent female predominance in subclinical strain abnormality contrasts with Duus et al., in which male sex independently predicted abnormal cardiac function; the discordance, likely reflecting differing cohorts, endpoints, and eras, remains an open question. Critically, LV dysfunction in this cohort did not correlate with lung function, suggesting a mechanism independent of pulmonary disease severity [44].

LV diastolic dysfunction appears to be an even more consistent finding. Koelling et al. compared 40 adults with advanced CF to patients with non-CF bronchiectasis and healthy controls using radionuclide ventriculography. Fractional LV filling at 50% of diastole was significantly lower in CF (54 ± 13%) than in bronchiectasis (66 ± 4%; *p* = 0.009) or controls (69 ± 14%; *p* = 0.0002), with a correspondingly greater contribution of atrial systole to total filling [68]. This impaired LV distensibility was specific to CF and not simply a consequence of chronic suppurative lung disease. Johnson et al. demonstrated that shifts in LV diastolic filling patterns were detectable early in the disease course and correlated directly with worsening pulmonary disease [70].

In the most comprehensive contemporary echocardiographic study, Duus et al. assessed 104 people with CF (93% on CFTR modulator therapy) matched 1:1 with population controls. LV diastolic function was worse in CF, and both LV and RV systolic function remained significantly impaired after multivariable adjustment [19]. These biventricular alterations persisted despite near-universal HEMT use, raising the question of whether established myocardial changes are only partially reversible, or whether CFTR modulation incompletely addresses the cardiac phenotype.

#### 3.2.2. Clinical Implications

The weight of evidence supports a model in which LV involvement arises from the convergence of ventricular interdependence and mechanical compression from RV overload, chronic hypoxemia and systemic inflammation driving myocardial injury and fibrosis, and intrinsic CFTR dysfunction in cardiomyocytes altering contractile physiology [7,12,14]. The detection of biventricular dysfunction in 44% of a contemporary, modulator-treated cohort suggests that cardiac involvement is neither rare nor fully preventable with current therapies [19]. Because this cohort is cross-sectional, it cannot distinguish pre-modulator remodeling from ongoing or treatment-resistant injury. As the CF population ages and encounters traditional cardiovascular risk factors, the interaction between CFTR-related myocardial vulnerability and acquired cardiac disease may become increasingly relevant. Longitudinal studies incorporating serial advanced imaging and correlation with clinical outcomes are needed to determine whether subclinical LV dysfunction progresses to clinically meaningful heart failure and whether early intervention can alter this trajectory. Whether CF cardiomyopathy persists, progresses, or can be prevented in patients who initiate HEMT early in life, before significant myocardial remodeling has occurred, is among the most important unanswered questions in CF cardiovascular medicine.

### 3.3. Electrophysiology and Arrhythmias

#### 3.3.1. Electrical Remodeling

CFTR functions as a cAMP-dependent chloride channel in the heart, where it modulates action potential duration (APD) and resting membrane potential. Levesque et al. first demonstrated CFTR mRNA expression in human atrium and mammalian ventricle, while Nagel et al. showed that the PKA-regulated cardiac chloride channel is functionally identical to epithelial CFTR, with a single-channel conductance of ~12 pS [22,71]. Under β-adrenergic stimulation, CFTR-mediated chloride efflux shortens APD, counterbalancing the prolonging effect of enhanced L-type calcium current, a critical mechanism for maintaining repolarization reserve during sympathetic activation [71,72].

Loss of CFTR disrupts this balance. As discussed in the Molecular Basis Section, CFTR-knockout cardiomyocytes require compensatory CaMKII and calcium-activated chloride channel (CaCC) activation to maintain contraction rate—a maladaptive dependency that increases susceptibility to calcium-mediated arrhythmogenesis [28]. Conversely, Ye et al. showed that transgenic CFTR overexpression in mice produced stress-related sudden death, high-grade AV block following isoproterenol, and easily inducible ventricular tachycardia on programmed stimulation [31]. Together, these gain- and loss-of-function models establish that precise CFTR dosage is critical for electrical stability, and that deviation in either direction creates an arrhythmogenic substrate.

The transmural distribution of CFTR adds further complexity. Wong et al. demonstrated a normal epicardial-to-endocardial gradient of CFTR expression in the LV that is lost in hypertrophied hearts, reverting to an embryonic pattern [73]. This loss of transmural repolarization heterogeneity—analogous to disruption of the potassium channel gradients implicated in long QT syndrome—is predicted to increase dispersion of repolarization and vulnerability to reentrant arrhythmias.

#### 3.3.2. Arrhythmogenic Triggers

The arrhythmia risk in CF is driven by a convergence of substrate and trigger factors operating across three domains:

Hypoxemia and exercise: Ruf and Hebestreit performed incremental exercise testing with continuous ECG monitoring in 75 CF patients and identified ventricular arrhythmias in 5 patients (6.7%), with significant exercise-induced desaturation (SpO_2_ ≤ 90%) occurring in 17 patients (23%)—though no single resting parameter reliably predicted either event [74].

Electrolyte disturbances: CF predisposes to hypokalemia, hyponatremia, and hypochloremic metabolic alkalosis through excessive sweat chloride losses [75,76]. Hypomagnesemia affects more than half of patients with advanced disease, driven by malabsorption, CFRD, and renal wasting from aminoglycosides [77]. The combination of hypokalemia and hypomagnesemia is particularly arrhythmogenic, lowering the threshold for both early and delayed afterdepolarizations.

Polypharmacy: CF patients are routinely exposed to multiple QT-prolonging agents, including chronic azithromycin, fluoroquinolones, and antifungals [78]. A significant association between chronic azithromycin and clinically meaningful QTc prolongation are limited—Avedissian et al. found no difference in median QTc between azithromycin users and non-users (405 vs. 394 msec; *p* = 0.14), and Magaret et al. confirmed safety in a pediatric trial [79,80,81]. However, borderline or prolonged QTc was present in ~20% of azithromycin-treated adults, particularly those on concomitant QT-prolonging medications, warranting continued vigilance [79,80].

Autonomic dysregulation, including sympathetic overactivity and vagal withdrawal documented in pediatric CF (see Special Populations), provides a further arrhythmogenic dimension [82].

#### 3.3.3. Atrial Remodeling and Atrial Fibrillation

Atrial fibrillation (AF) was identified as a component of MACEs in the Frost et al. multinational cohort [9]. Bhat et al. found that primary cardiac admissions—predominantly AF, heart failure, and myocardial infarction—increased by 16.4% per year (IRR 1.16; 95% CI 1.04–1.29; *p* = 0.009), with cardiac admissions associated with higher unadjusted mortality (OR 3.70; 95% CI 1.61–8.53) [10].

The structural substrate for AF has been characterized by Dordevic et al., who performed strain echocardiography in 82 CF patients and 32 matched controls. Both left and right atrial volume indices were significantly increased in CF, with LA reservoir and conduit function significantly impaired [83]. LA and RA enlargement predicted mortality in univariate analysis, though only FVC remained independently predictive after multivariable adjustment [83]. This biatrial remodeling—combining chamber dilation with functional impairment—provides the anatomic and electrophysiological substrate for AF, consistent with the atrial cardiomyopathy paradigm.

#### 3.3.4. Sudden Cardiac Death

Sudden cardiac death in CF, though rare, has been documented in two contexts. The first is the infantile cardiomyopathy phenotype described by Zebrak et al., in which myocardial fibrosis and necrosis presented as cardiac arrest in children under 2 years, predominantly in F508del homozygotes, discussed in the LV and Biventricular Section [33]. The second is exercise- or stress-related death in older patients with advanced disease, where the combination of hypoxemia, electrolyte derangement, autonomic dysfunction, and a structurally remodeled myocardium creates a substrate for lethal ventricular arrhythmias. The Ye et al. transgenic model provides mechanistic insight: sudden death occurred exclusively under adrenergic stress, suggesting that perturbation of cardiac chloride channel activity—whether by excess or deficiency—can be lethal when combined with sympathetic activation [31].

Sullivan et al. described recurrent supraventricular tachycardia in CF patients with cor pulmonale, noting that the interaction of hypoxia, infection, intensive bronchodilator therapy, and altered autonomic responses likely precipitated arrhythmia [84]. As the CF population ages and accumulates both disease-specific and traditional cardiovascular risk factors, systematic arrhythmia surveillance, e.g., exercise testing with ECG monitoring and periodic Holter assessment in higher-risk patients, may warrant prospective evaluation, but is not currently guideline-endorsed.

### 3.4. Valve and Pericardial Manifestations

#### 3.4.1. Tricuspid Valve Disease

Tricuspid regurgitation (TR) is the most clinically relevant valvular abnormality in CF, arising predominantly as functional (secondary) regurgitation from RV pressure overload and annular dilation. In early echocardiographic studies, TR was infrequent in patients with moderate disease. Panidis et al. found only 1 of 17 CF patients with mild TR by Doppler [85]. However, in end-stage disease, TR becomes far more prevalent. Belle-van Meerkerk et al. reported a measurable TR jet in 67% of 93 CF patients evaluated for transplantation, with echocardiographic estimation of systolic pulmonary artery pressure from the TR jet achieving a negative predictive value of 96% for excluding pulmonary hypertension when the estimated sPAP was low [86]. Scaravilli et al. confirmed the hemodynamic substrate for progressive functional TR in transplant candidates, demonstrating increased RV end-diastolic area and wall thickness with subnormal TAPSE, tissue Doppler systolic velocity, and fractional area change [87].

The clinical significance of TR in CF extends beyond its role as a hemodynamic marker. Progressive TR begets further RV dilation through volume overload, creating a self-perpetuating cycle that accelerates RV failure. Whether TR carries independent prognostic significance in CF—where the driver is pulmonary vascular disease rather than intrinsic valve pathology—remains to be established. In the general population, moderate or greater TR is independently associated with increased mortality regardless of pulmonary artery pressure or LV ejection fraction, but this association has not been demonstrated in CF [86].

#### 3.4.2. Left-Sided Valvular Considerations

Primary left-sided valvular disease has not been identified as a characteristic feature of CF. Mitral valve prolapse, which occurs in 2–3% of the general population and is associated with connective tissue disorders and thoracic skeletal abnormalities such as pectus excavatum, has been anecdotally reported in CF patients who share these body habitus features, but no systematic echocardiographic study has demonstrated an increased prevalence [88,89]. Aortic valve disease has not been reported in CF.

#### 3.4.3. Pericardial Disease

Pericardial involvement in CF was historically considered rare, limited to case reports of purulent pericarditis as a complication of contiguous pulmonary infection. Altemeier et al. described *Pseudomonas aeruginosa* purulent pericarditis arising from direct extension of chronic lower respiratory tract infection, and Downes et al. subsequently reported non-typeable *Haemophilus influenzae* purulent pericarditis in a child with CF [90,91]. These cases underscore that the anatomic proximity of infected airways to the pericardium, combined with the virulence of CF-associated pathogens, creates a unique risk for suppurative pericardial disease—a complication whose clinical presentation may be masked by the underlying respiratory illness.

A novel single-center observation from Rodriguez-Takeuchi et al. systematically evaluated pericardial calcification on chest CT in 348 adult CF patients; as a first-in-kind finding, it requires independent replication before it can be considered an established prevalence [92]. Pericardial calcification was present in 18% of CF patients—strikingly higher than in age- and sex-matched controls without CF (1%), individuals with prior tuberculosis (2%), and patients with primary ciliary dyskinesia (4%) (*p* < 0.001) [92]. The calcification was most frequently multifocal (58%), developed de novo in 66% of affected patients, and progressed over time in 21%. Older age, lower FEV_1_, higher vitamin D levels, and *Burkholderia cenocepacia* ET12 infection were independently associated with pericardial calcification [92].

These findings are significant for several reasons. First, the 18% prevalence is remarkably high for a finding traditionally associated with tuberculosis, prior cardiac surgery, or radiation—none of which are common in CF. Second, the association with *B. cenocepacia* ET12, a pathogen known for its capacity to cause invasive disease and necrotizing pneumonia, suggests that chronic pericardial inflammation from contiguous infection may be a more common subclinical process than previously appreciated. Third, the progressive nature of the calcification raises the question of whether a subset of CF patients may develop constrictive physiology over time, particularly as they age. The clinical significance of pericardial calcification in CF, specifically whether it is associated with diastolic impairment, exercise intolerance, or adverse outcomes, remains to be determined and represents an important area for future investigation.

#### 3.4.4. Catheter-Related Valvular Complications

CF patients frequently require long-term central venous access for recurrent courses of intravenous antibiotics. Aitken and Tonelli reviewed 87 indwelling catheters in 65 CF patients over a 10-year period and reported an overall complication rate of 0.463 per 1000 catheter-days, with thrombosis (n = 14) and infection (n = 9) as the most common complications [93]. Cargill et al. found that 82% of clinically significant bloodstream infections in CF originated from totally implantable vascular access devices, with coagulase-negative *staphylococci* and *Candida species* as the predominant organisms [94]. May et al. confirmed that poor nutritional status, *B. cepacia* complex infection, and multiple line insertions were independent risk factors for catheter complications [95].

While frank infective endocarditis from catheter-related bacteremia remains rare in CF, the combination of chronic indwelling catheters, recurrent bacteremia, and potential right-sided valvular damage from catheter trauma creates a theoretical risk that may become more relevant as patients survive longer. Systematic surveillance for catheter-related endocarditis has not been performed in CF cohorts, representing a knowledge gap warranting attention.

### 3.5. Coronary Artery Disease

The vascular pathobiology underlying coronary risk in CF, including endothelial dysfunction, arterial stiffness, preclinical atherosclerosis phenotypes, and chronic inflammatory endothelial activation, is detailed in Section 2.6.

Coronary artery disease (CAD) has historically been considered rare in CF, attributed to the traditionally low BMI, favorable lipid profiles, and shortened lifespan of affected individuals. However, emerging data challenge this assumption. In the Frost et al. multinational cohort, myocardial infarction was a component of MACEs, with CF conferring a 65% higher risk than the general population [9]. Bhat et al. identified MI among the primary cardiac admission diagnoses, with increasing cardiac admissions (Section 3.3) [10]. Case reports have documented acute STEMI in young women with CF and CFRD, underscoring the importance of considering myocardial ischemia in the differential diagnosis of chest pain in CF [96].

Multiple mechanistic pathways predispose CF patients to accelerated atherogenesis. Chronic systemic inflammation, endothelial dysfunction from childhood, and direct CFTR-mediated effects on vascular smooth muscle, including enhanced neointimal formation and exaggerated vasoconstriction, collectively create a pro-atherogenic milieu [30,52,53,59]. The divergent preclinical atherosclerosis phenotypes identified by Nowak et al., functional stiffening in pancreatic-insufficient patients versus structural intimal thickening in pancreatic-sufficient patients, suggest that the pathways to coronary disease may differ by CF phenotype [57].

#### The Metabolic Shift in the HEMT Era

The nutritional landscape of CF is undergoing a fundamental transformation. In the HEMT era, the prevalence of overweight (BMI 25–29.9 kg/m^2^) has increased by over 300% and obesity (BMI > 30 kg/m^2^) by over 400% in CF Foundation Patient Registry data [97]. Overweight CF patients demonstrate higher systolic blood pressure, higher LDL cholesterol, and greater insulin resistance compared with normal-weight and underweight counterparts [20]. Higher visceral adiposity, even at normal BMI (“normal-weight obesity”), has been documented in CF and inversely correlates with lung function [2]. Gramegna et al. demonstrated that 6 months of ETI therapy increased cholesterol, triglycerides, and apolipoprotein-B levels while decreasing inflammatory markers, a mixed cardiovascular risk profile [51]. The CF Foundation position paper acknowledges that the legacy high-fat, high-calorie diet may carry long-term cardiovascular consequences as patients achieve normal or supranormal nutritional status on HEMT [15].

Post-transplant CAD, driven by chronic immunosuppression, is discussed in the Management Section.

### 3.6. Heart Failure

Heart failure in CF encompasses a spectrum from subclinical biventricular dysfunction to overt clinical decompensation, with the dominant phenotype evolving across the pre-HEMT and post-HEMT eras.

#### 3.6.1. Pre-HEMT Era

In the pre-modulator era, heart failure in CF was predominantly right-sided, representing the terminal manifestation of cor pulmonale. Progressive pulmonary disease led to chronic hypoxemia, pulmonary vascular remodeling, and rising pulmonary vascular resistance, ultimately exhausting RV compensatory mechanisms, a trajectory detailed in the RV Dysfunction Section [46,47]. Left-sided heart failure was considered rare, though subclinical LV diastolic dysfunction was demonstrable even in early disease; Koelling et al. showed impaired LV filling specific to CF and not simply a consequence of chronic suppurative lung disease, as discussed in the LV and Biventricular Section [68]. The Ramsey et al. analysis of national hospitalization trends (2002–2017) documented that congestive heart failure was more than 10 times more prevalent among adult CF hospitalizations compared with pediatric admissions, reflecting the emerging burden as patients survived longer [98].

#### 3.6.2. Post-HEMT Era

HEMT has dramatically reduced the pulmonary drivers of RV failure, with lung transplant listings decreasing to approximately 20% of pre-ETI levels [99]. However, heart failure has not disappeared from the CF landscape. Left-sided heart failure was a component of MACEs in the Frost et al. multinational analysis, with CF conferring significantly higher risk than the general population [9]. Bhat et al. identified heart failure among the primary cardiac admission diagnoses, with increasing cardiac admissions (Section 3.3) [10]. Critically, Duus et al. demonstrated that 44% of a contemporary, modulator-treated cohort had abnormal cardiac function (defined and discussed in Section 2.1) [19]. These findings, combined with the diffuse interstitial fibrosis documented on cardiac MRI by Lagan et al., raise the possibility that established myocardial remodeling may be only partially reversible with CFTR modulation [13].

The emerging heart failure phenotype in the post-HEMT era may increasingly resemble HFpEF, driven by the convergence of myocardial fibrosis, diastolic dysfunction, hypertension from corrected salt-wasting, obesity, and CFRD. Whether CF should be recognized as a distinct etiology of HFpEF, analogous to other systemic inflammatory conditions, warrants investigation. Given all the cardiovascular manifestations, Table 1 describes the cardiovascular screening approaches (described in Section 5.3) in CF, and Table 2 describes the summary of the cardiovascular manifestations of CF.

## 4. Special Populations

### 4.1. Pediatric Population

Cardiovascular alterations in CF are not confined to adults—they are detectable from early childhood, often preceding clinically significant pulmonary disease. Eising et al. demonstrated that children aged 3–12 years with CF and normal lung function had significantly reduced global LV and RV strain compared with healthy controls, even after adjustment for lung function, though residual confounding cannot be excluded, alongside elevated pulse wave velocity (5.76 ± 0.57 vs. 5.43 ± 0.61 m/s; *p* = 0.049) [45]. Kartal Öztürk et al. confirmed increased arterial stiffness in a larger pediatric cohort, with an augmentation index approximately 8% higher in CF children, corresponding to roughly 10 years of vascular aging, despite the absence of traditional cardiovascular risk factors [61]. Kreslová et al. showed that biomarkers of endothelial activation (E-selectin, VCAM-1, hsCRP) are significantly elevated from childhood, preceding the functional decline in the reactive hyperemia index that becomes apparent in adulthood, a temporal sequence suggesting a window for early intervention [60].

Autonomic dysregulation is also present early. Heart rate variability studies in children with CF demonstrate sympathetic overactivity and vagal withdrawal, particularly in those colonized with Pseudomonas aeruginosa, mirroring the autonomic profile associated with increased arrhythmia risk in other chronic cardiopulmonary diseases [82]. In the pediatric inpatient setting, PH during acute exacerbations has been identified in approximately 24% of hospitalized children [100].

These findings collectively establish that the cardiovascular consequences of CF begin in childhood across multiple domains, myocardial mechanics, vascular stiffness, endothelial function, and autonomic regulation, and support the rationale for early cardiovascular surveillance, particularly as HEMT is now initiated in infancy.

### 4.2. Heart–Lung vs. Isolated Lung Transplantation

The choice between bilateral lung transplantation (BLTx) and combined heart–lung transplantation (HLTx) in CF has been debated. Ganesh et al. analyzed 181 CF transplants in the UK (88 BLTx, 93 HLTx) and found no significant difference in adjusted survival (HR 0.77; 95% CI 0.29–2.06), time to first rejection, or infection rates [101]. Vricella et al. reported similar results from the Stanford experience (42 BLTx, 22 HLTx), with actuarial survival of 93.2% at 1 year and 61.8% at 5 years regardless of procedure type; notably, 82% of hearts from HLTx recipients were used as domino heart transplants, ameliorating the impact on organ availability [102].

However, Kitai et al. provided a contrasting long-term perspective from the UNOS database (98,310 transplants, 1987–2016), demonstrating that while HLTx carried higher early postoperative mortality, long-term adjusted mortality was significantly lower beyond 3 years compared with isolated lung transplantation, with reduced rates of bronchiolitis obliterans syndrome and coronary allograft vasculopathy—suggesting immunoprotective benefits of combined organ transplantation [103]. These data support consideration of HLTx in CF patients with significant cardiac involvement, though the decision must be individualized based on the degree of cardiac dysfunction, organ availability, and institutional expertise.

A systematic review and meta-analysis by Yan et al. comparing double-lung versus heart–lung transplantation for end-stage cardiopulmonary disease similarly found no definitive survival advantage for either approach, though the analysis was limited by heterogeneity across indications and eras [104].

## 5. Diagnostic and Screening Strategies

### 5.1. Biomarkers

No validated cardiovascular risk biomarker panel specific to CF has been established to date [7]. Several circulating markers of endothelial activation, including sICAM-1, sE-selectin, vWF, tissue-plasminogen activator, P-selectin, and sVCAM-1, are significantly elevated in CF and correlate with disease severity and inflammatory activity [48,49,50]. De Rose et al. showed that sICAM-1 and sE-selectin inversely correlated with FEV_1_, increased during pulmonary exacerbations, and declined after antibiotic therapy, linking endothelial activation to the inflammatory cycle [48]. Romano et al. demonstrated that inflammatory cytokines (TNF-α, IL-6) correlated directly with endothelial injury markers, supporting a close interaction between chronic inflammation and vascular dysfunction [49]. The significance of elevated sVCAM-1 as a possible marker of CFTR-related microangiopathy, based on a single cross-sectional cohort, is discussed in Section 2.6 [50].

NT-proBNP has limited sensitivity in CF; population-level values do not differ from controls, though a subset (~25%) show mild elevations that may signal subclinical cardiac stress [67].

### 5.2. Multimodality Imaging

Conventional transthoracic echocardiography (TTE) remains the first-line imaging tool, but its sensitivity for detecting subclinical disease is limited. Speckle-tracking strain echocardiography has proven superior: LV global longitudinal strain (GLS) and strain rate are reduced in CF patients even when LVEF is normal, with one study identifying decreased LV strain rates in 100% of CF patients despite normal ejection fractions [44,69]. RV free wall strain is similarly impaired in clinically stable patients without overt pulmonary hypertension and correlates with spirometric FEV_1_ [62]. Serial echocardiographic parameters—including TAPSE, RV strain, and the TAPSE:PASP ratio as a noninvasive surrogate for RV–PA coupling—offer the most practical approach for longitudinal surveillance [40,41,62].

Importantly, the utility of advanced echocardiographic techniques for detecting subclinical cardiac disease persists in the post-HEMT era. Duus et al. assessed 104 people with CF, 93% receiving CFTR modulator therapy, matched 1:1 with population controls, and found that echocardiographic assessment identified abnormal cardiac function in 44% of CF patients, more than double the prevalence in controls (defined and discussed in Section 2.1) [19]. These data confirm that echocardiographic screening, particularly with tissue Doppler and strain imaging, remains clinically relevant in the modulator era and should not be deferred on the assumption that HEMT normalizes cardiac function.

Cardiac MRI (CMR) provides unique advantages through multiparametric tissue characterization. T1 mapping and extracellular volume (ECV) imaging detect diffuse myocardial fibrosis, the predominant driver of LV hypertrophy in stable CF, while T2 mapping identifies myocardial edema during acute exacerbations, with edema severity correlating strongly with airflow limitation [13]. CMR is also the reference standard for RV volumetric assessment, which is particularly relevant given the complex RV geometry that limits echocardiographic accuracy.

No dedicated cardiac MRI study has yet been performed exclusively in a post-HEMT cohort. Whether the diffuse myocardial fibrosis and extracellular volume expansion demonstrated by Lagan et al. are reversible with sustained CFTR modulation remains an important unanswered question and represents a priority for future investigation [13].

**Figure 2:**
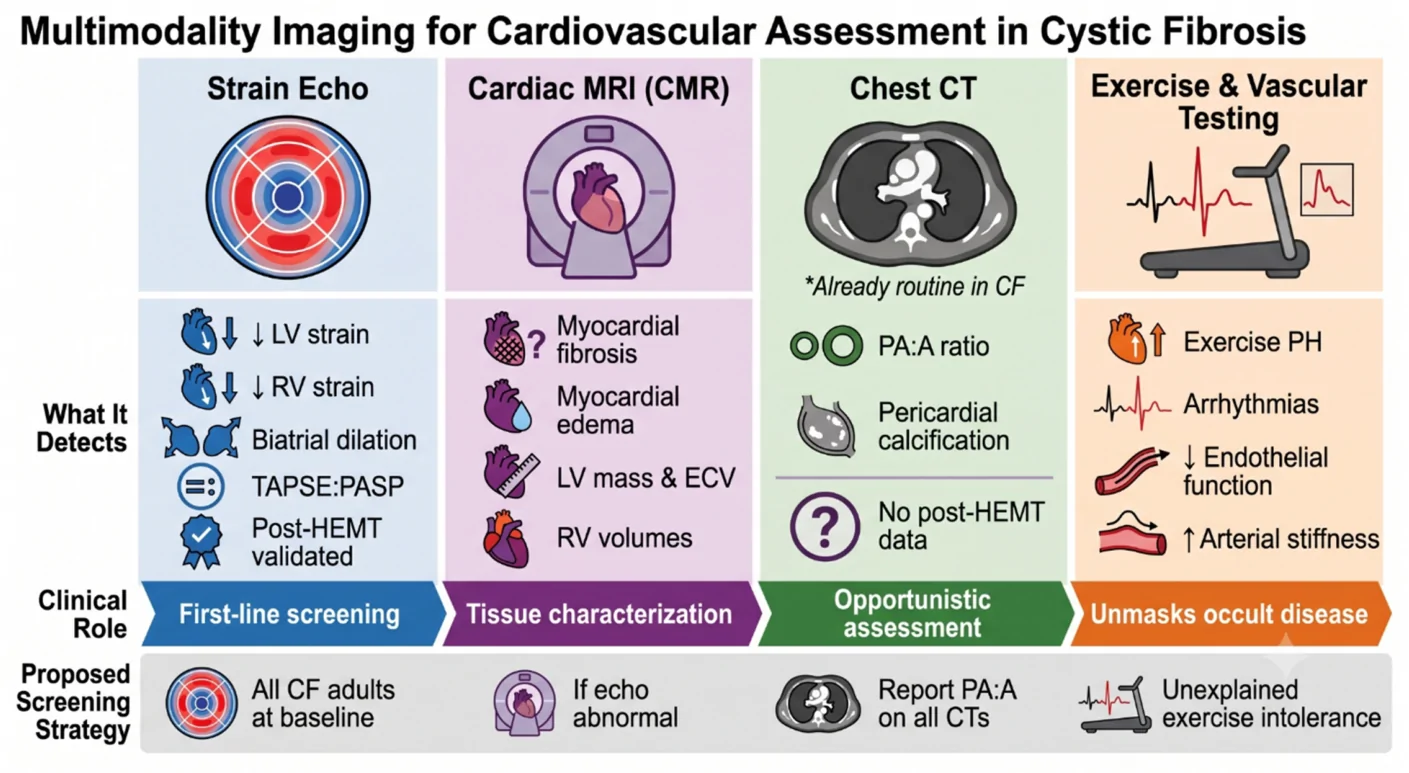
Multimodality Imaging for Cardiovascular Assessment in Cystic Fibrosis. Four imaging modalities are summarized by what they detect, their clinical role, and an author-proposed screening strategy. The bottom (‘Proposed Screening Strategy’) row represents author-proposed, research-stage suggestions that are not endorsed by current guidelines and require prospective, outcome-based validation; universal baseline strain echocardiography in particular is not a guideline recommendation. The 18% pericardial calcification prevalence shown derives from a single-center cohort and awaits independent replication. Strain echocardiography serves as first-line screening with post-HEMT validation; cardiac MRI provides tissue characterization (fibrosis, edema) but lacks post-HEMT-specific data; chest CT offers opportunistic assessment of the PA:A ratio and pericardial calcification; and exercise/vascular testing unmasks occult disease not apparent at rest. Abbreviations: CMR, cardiac magnetic resonance; CT, computed tomography; ECV, extracellular volume fraction; HEMT, highly effective modulator therapy; LV, left ventricular; PA:A, pulmonary artery-to-aorta ratio; PASP, pulmonary artery systolic pressure; PH, pulmonary hypertension; RV, right ventricular; TAPSE, tricuspid annular plane systolic excursion.

Chest CT, already performed routinely for pulmonary surveillance, provides opportunistic cardiovascular information. The pulmonary artery-to-aorta (PA:A) ratio serves as a practical screening tool for pulmonary vascular disease: a PA:A ratio > 1 independently predicts both PH on catheterization (adjusted OR 4.50) and death or transplant (adjusted HR 2.69), and is associated with acute pulmonary exacerbations even after adjustment for other risk factors [65,66]. Pericardial calcification, reported in 18% of CF adults in a single-center chest CT cohort (replication pending), represents an additional incidental finding warranting recognition, as discussed in the Valve and Pericardial Section [92]. Figure 2 summarizes the multimodality imaging modalities.

### 5.3. Proposed Screening Intervals

No formal, universally adopted screening intervals for cardiac disease in CF exist. Current recommendations are drawn from multiple sources:

Echocardiography: In the context of lung transplant referral, the CF Foundation consensus guidelines recommend a baseline echocardiogram to screen for pulmonary hypertension in adults (≥18 years) with FEV_1_ < 40% predicted and in children with FEV_1_ < 50% predicted; this threshold was defined for pre-transplant evaluation rather than as a general cardiac-screening program [105].

Diabetes: Oral glucose tolerance testing starting at age 10, as HbA1c and fructosamine are inaccurate in CF [106].

Lipid screening: A fasting lipid profile at least once between ages 10 and 20, repeated every 4–6 years thereafter, with more frequent monitoring in individuals with multiple cardiovascular risk factors [107].

Blood pressure: Measured at least annually during clinic visits in children and adolescents aged 3–17 years [11].

These thresholds were established before the widespread adoption of HEMT and the recognition of subclinical biventricular dysfunction in modulator-treated patients [19]. Whether screening echocardiography should be extended to all CF adults, regardless of FEV_1_, and whether advanced imaging (strain echocardiography, CMR) should be incorporated into routine surveillance are questions that require prospective evaluation.

Implementation considerations: Strain echocardiography and cardiac MRI require specialized acquisition and analysis expertise that is not uniformly available even in well-resourced CF centers, and no proposed strategy has been validated against clinical outcomes or evaluated for cost-effectiveness. Any expansion of cardiac surveillance would need to be operationalized through CF care networks or national registries and prospectively assessed before adoption. In particular, universal baseline strain echocardiography would require echocardiographic strain capability and reader expertise beyond that available at many CF centers, and its adoption should be contingent on prospective, outcome-based and cost-effectiveness validation, ideally embedded in national CF registries.

## 6. Management and Therapeutic Implications

### 6.1. Optimization of Pulmonary Disease to Reduce RV Load

The cornerstone of cardiovascular management in CF remains aggressive treatment of the underlying lung disease. Pulmonary hypertension in CF is predominantly WHO Group 3 (PH due to lung disease and/or hypoxia), and its severity tracks closely with the degree of airflow obstruction and hypoxemia [108]. Optimization of airway clearance, antimicrobial therapy, nutritional support, and supplemental oxygen constitutes the primary strategy for reducing pulmonary vascular resistance and RV afterload.

Supplemental oxygen therapy is indicated for resting or exercise-induced hypoxemia; by analogy with COPD, it is expected to attenuate hypoxic pulmonary vasoconstriction and slow PH progression, though CF-specific randomized data are lacking [109]. Non-invasive ventilation may further reduce RV afterload in patients with advanced CF lung disease (ACFLD) by improving alveolar ventilation and correcting hypercapnia [110,111].

The role of PH-specific pharmacotherapy remains uncertain. Small studies of sildenafil have demonstrated safety in mild-to-moderate CF lung disease, with improvements in exercise capacity, vascular endothelial function (FMD increased by 1.5% after 4 weeks), and right heart function on cardiac MRI [112,113,114]. However, one study noted a decline in FEV_1_ during sildenafil treatment despite improved exercise tolerance, raising concerns about ventilation–perfusion mismatch [112]. Citing insufficient evidence, the 2020 ACFLD guidelines do not provide a recommendation regarding PH-specific therapy in CF [111]. The 2022 ESC/ERS PH guidelines similarly caution against extrapolating Group 1 PAH treatment paradigms to Group 3 PH, where pulmonary vasodilators may worsen gas exchange [115]. In practice, PH-targeted therapy is reserved for selected patients with disproportionate PH relative to lung disease severity, ideally confirmed by right heart catheterization and managed in collaboration with a PH specialist.

For refractory RV failure, general principles apply: judicious diuresis to optimize preload, avoidance of systemic hypotension, inotropic support (dobutamine or milrinone) in the acute setting, and avoidance of invasive mechanical ventilation whenever possible due to the high risk of hemodynamic deterioration [109,110].

### 6.2. CFTR Modulator Therapy: Cardiovascular Implications

HEMT—particularly ETI—is expected to attenuate the pulmonary vascular remodeling and chronic hypoxemia that drive RV dysfunction. No study has directly measured the effect of HEMT on pulmonary artery pressure or RV function. The dramatic reduction in lung transplant listings reflects improved overall pulmonary status but cannot, by itself, establish that HEMT reduces pulmonary vascular remodeling or RV afterload; this requires longitudinal pre/post-HEMT hemodynamic and imaging studies [99].

However, HEMT does not fully address the cardiac phenotype. As discussed in the LV and Biventricular Section, Duus et al. found that 44% of patients on modulator therapy had abnormal cardiac function (defined and discussed in Section 2.1) [19]. This underscores the importance of early modulator initiation and the need for dedicated cardiac outcome studies.

HEMT also introduces new cardiovascular considerations through three mechanisms.

Hypertension: ETI corrects the salt-wasting phenotype by improving NaCl and fluid conservation, leading to increased blood pressure, decreased aldosterone levels, and normalization of plasma sodium [116]. In phase 3 trials, mean systolic and diastolic blood pressure increased by 3.0 and 2.5 mmHg, respectively, with ETI compared to active controls [117]. Post-marketing surveillance has identified cases of new-onset hypertension requiring antihypertensive therapy within weeks of ETI initiation [118]. The Alyftrek (vanzacaftor/tezacaftor/deutivacaftor) label similarly notes blood pressure elevations, with 3.5% of treated patients developing systolic BP > 140 mmHg with >10 mmHg increase from baseline on at least two occasions [119]. Berg et al. have proposed that salt repletion—a longstanding recommendation in CF care—may be unnecessary in ETI-treated patients and could contribute to hypertension risk [116].

Dyslipidemia: Gramegna et al. demonstrated that 6 months of ETI therapy increased cholesterol, triglycerides, and apolipoprotein-B levels while decreasing inflammatory markers—a mixed cardiovascular risk profile [51]. The CF Foundation position paper recommends lipid screening following general population guidelines until CF-specific data become available [15].

Weight gain and metabolic shift: The prevalence of overweight (BMI 25–29.9) has increased by over 300% and obesity (BMI > 30) by over 400% in CF Foundation Patient Registry data in the HEMT era [97]. Overweight CF patients demonstrate higher systolic blood pressure, higher LDL cholesterol, and greater insulin resistance compared with normal-weight counterparts [20]. The traditional high-fat, high-calorie, high-salt diet—developed for an era of malabsorption—may now carry long-term cardiovascular consequences as patients achieve normal or supranormal nutritional status on HEMT [15,120].

### 6.3. Drug–Drug Interactions with Cardiovascular Medications

ETI is both a substrate and a perpetrator of clinically relevant interactions that matter as CF patients acquire cardiovascular comorbidities. ETI is a CYP3A substrate: strong CYP3A inducers (e.g., rifampin, carbamazepine, phenytoin) are not recommended, and moderate-to-strong CYP3A inhibitors require ETI dose reduction [121].

As a perpetrator, elexacaftor and its M23 metabolite inhibit OATP1B1/1B3, raising exposure of statins; physiologically based pharmacokinetic modeling predicts moderate interactions for atorvastatin (AUC ratio 3.27), pravastatin (2.27), and pitavastatin (2.24), with only a weak interaction for rosuvastatin (1.83), favoring rosuvastatin and statin dose reduction with monitoring for myopathy [122,123].

Ivacaftor inhibits CYP2C9, so INR monitoring is recommended with warfarin [123]. For direct oral anticoagulants, often needed for CF-related atrial fibrillation and complicated by baseline hemoptysis risk, the relevant axis is P-glycoprotein: ivacaftor is a weak P-gp inhibitor (digoxin AUC increased 1.3-fold), so caution and monitoring are advised with narrow-therapeutic-index P-gp substrates [123].

Notably, clinical data temper the theoretical CYP3A-inhibitor concern, as tezacaftor/ivacaftor was not a clinical CYP3A inhibitor, so interaction risk should not be overstated [123].

### 6.4. Lifestyle and Cardiovascular Risk Modification

Exercise remains a critical therapeutic modality with direct cardiovascular benefits. Current recommendations advise 30–60 min of moderate-intensity aerobic exercise at least 4 days per week [124]. Anifanti et al. demonstrated in a randomized controlled trial that a 1-year wearable activity tracker-based exercise program significantly improved RV systolic function (TAPSE increased by 8.3%, RV free-wall longitudinal strain by 13.7%), RV diastolic function (TVE/A ratio increased by 13.2%), and reduced estimated pulmonary artery systolic pressure by 7.6%—with a strong inverse correlation between daily step count and PASP (r = −0.727; *p* < 0.01) [125]. These data provide the first direct evidence that structured exercise can favorably remodel the right heart in CF.

Cardiovascular risk stratification should incorporate both traditional and disease-specific factors. The Frost et al. multinational study identified age, diabetes, hypertension, lung function decline, and intravenous antibiotic days as independent predictors of MACEs, establishing that CF-specific disease burden compounds conventional cardiovascular risk [9]. Given the elevated MACE risk relative to SLE, RA, and HIV (Section 1), consideration of CF as a cardiovascular risk-enhancing condition, analogous to its classification in other systemic inflammatory diseases, is warranted [9].

### 6.5. Transplantation Considerations

Bilateral lung transplantation (BLTx) remains the definitive surgical option for end-stage CF lung disease, with median post-transplant survival approaching 10 years [126]. The CF Foundation consensus guidelines recommend transplant referral when FEV_1_ declines below 40% predicted, with earlier referral for patients with PH, supplemental oxygen requirement, hypercarbia, or BMI < 18 [105].

The choice between BLTx and combined heart–lung transplantation (HLTx) is discussed in the Special Populations Section, where the Ganesh et al., Vricella et al., and Kitai et al. data are presented [101,102,103]. In brief, short-term survival is comparable between approaches, but long-term data suggest potential immunoprotective benefits of HLTx beyond 3 years, supporting its consideration in CF patients with significant cardiac involvement.

HEMT has dramatically reduced the need for transplantation, but continuation of CFTR modulators post-transplant for extrapulmonary indications (sinusitis, gastrointestinal symptoms, CFRD) is an evolving practice, with approximately one-third of recipients discontinuing therapy due to side effects or lack of perceived benefit [2]. Post-transplant cardiovascular surveillance is essential, as chronic immunosuppression drives accelerated atherosclerosis: in a Dutch cohort of 100 CF lung transplant recipients, macrovascular disease prevalence rose to 15.9% by 15 years post-transplant, with diabetes increasing from 63% at transplant to over 90% and dyslipidemia from 21% to over 80% [17,18].

## 7. Future Directions and Conclusions

### 7.1. Future Directions

Several critical knowledge gaps must be addressed to advance the cardiovascular care of people with CF.

#### 7.1.1. Natural History in the Post-HEMT Era

The most pressing need is prospective, longitudinal cardiac phenotyping of CF cohorts receiving HEMT. Whether the 44% prevalence of abnormal cardiac function in modulator-treated patients represents irreversible pre-modulator remodeling or ongoing CFTR-mediated cardiac injury remains unknown [19]. Multicenter registries incorporating serial echocardiography, cardiac MRI, and cardiac biomarkers (NT-proBNP, high-sensitivity troponin) are needed to determine whether early modulator initiation—particularly in infants and young children—can prevent the subclinical biventricular dysfunction and arterial stiffness already detectable in pediatric CF [45].

#### 7.1.2. Disentangling Primary from Secondary Cardiac Involvement

Whether CFTR dysfunction causes intrinsic cardiomyopathy independent of lung disease remains unresolved [12,14]. Answering this requires cardiac-specific conditional CFTR-knockout animal models, human induced pluripotent stem cell-derived cardiomyocyte studies, and careful clinical phenotyping of patients with preserved lung function but cardiac abnormalities. Multiparametric cardiac MRI, capable of simultaneously quantifying myocardial fibrosis (ECV), edema (T2 mapping), and ventricular function, offers a non-invasive pathway to characterize the CF myocardium in vivo [13].

#### 7.1.3. Cardiovascular Risk Stratification and Prevention

The elevated MACE risk relative to SLE, RA, and HIV (Section 1) demands the development of CF-specific cardiovascular risk prediction tools [9]. Machine learning approaches, already applied to CF prognostication using UK registry data, could integrate disease-specific variables (genotype, FEV_1_ trajectory, exacerbation frequency, CFRD status) with traditional cardiovascular risk factors to generate individualized risk scores [127]. Whether CF should be formally classified as a cardiovascular risk-enhancing condition, triggering earlier lipid screening, blood pressure monitoring, and lifestyle counseling, warrants consensus discussion among CF and cardiology societies. Finally, nearly all cited cohorts comprise European or North American, predominantly European-ancestry populations; the cardiovascular phenotype of CF in other ancestral groups is essentially uncharacterized and warrants dedicated study.

#### 7.1.4. Emerging Therapeutic Targets

Next-generation CFTR modulators (e.g., vanzacaftor/tezacaftor/deutivacaftor) and mutation-agnostic approaches including nucleic acid-based therapies (mRNA, antisense oligonucleotides, gene editing) may extend CFTR correction to the ~10% of patients ineligible for current modulators, a population at highest cardiovascular risk [128,129]. Whether these therapies also restore cardiac CFTR function is unknown. Additionally, the cardiometabolic consequences of HEMT—hypertension, dyslipidemia, obesity—represent modifiable targets that require CF-specific clinical trials of dietary modification, exercise prescription, and pharmacologic intervention [51].

#### 7.1.5. Technology-Enabled Monitoring

Wearable devices capable of continuous heart rate, rhythm, oxygen saturation, and activity monitoring could enable early detection of arrhythmias and exercise-induced desaturation in ambulatory CF patients. Integration of wearable data with electronic health records and AI-driven analytics may facilitate real-time cardiovascular risk surveillance [130].

### 7.2. Conclusions

Cardiovascular disease in CF extends far beyond the traditional paradigm of cor pulmonale. The evidence reviewed herein documents, across predominantly cross-sectional and single-center studies, biventricular systolic and diastolic dysfunction, myocardial fibrosis, endothelial dysfunction, accelerated arterial stiffness, electrical remodeling, and atrial and pericardial abnormalities, together with population-based data showing an elevated risk of MACEs comparable to established inflammatory conditions. Whether CFTR dysfunction independently causes clinically significant cardiomyopathy in humans remains unproven and is a priority for longitudinal study. CFTR modulator therapy has transformed the trajectory of CF, yet it introduces new cardiovascular considerations, including hypertension and dyslipidemia, while failing to fully reverse established cardiac remodeling. As the CF population ages into the fifth, sixth, and seventh decades of life, a paradigm shift is required: from reactive management of end-stage cardiopulmonary failure to proactive, systematic cardiovascular surveillance and risk modification integrated into routine CF care. Achieving this will demand collaboration between pulmonologists, cardiologists, and the broader CF multidisciplinary team, supported by dedicated research that places the heart at the center of the evolving CF narrative.

## Figures and Tables

**Table 1:** Evidence grading of cardiovascular screening approaches in CF.

Approach	Evidence Tier	Basis
Blood pressure at routine visits; OGTT from age 10; lipid screening ages 10–20	Guideline-supported	CF Foundation/nutrition guidelines [1]
Baseline echo for PH at FEV_1_ thresholds (pre-transplant context)	Guideline-supported (transplant referral)	CF Foundation consensus [1]
PA:A ratio on routine chest CT for PH risk	Observationally supported	Single-/multi-center cohorts
Strain echo/CMR for subclinical dysfunction in all adults	Author-proposed	Cross-sectional cohorts; no outcome validation
Holter/exercise ECG for arrhythmia surveillance	Author-proposed	Extrapolated; not validated in CF

This table summarizes the strength of evidence behind five cardiovascular screening strategies in adults with cystic fibrosis, sorting each into a tier—guideline-supported, observationally supported, or author-proposed. It lets a clinician see at a glance which screening practices rest on firm guideline footing versus which are emerging or speculative. Abbreviations: CF: Cystic fibrosis; CMR: Cardiac magnetic resonance imaging; CT: Computed tomography; ECG: electrocardiogram; Echo: Echocardiography; FEV: Forced expiratory volume; OGTT: Oral glucose tolerance test; PA:A: Pulmonary artery-to-aorta diameter ratio; PH: Pulmonary hypertension.

**Table 2:** Summary of Cardiovascular Manifestations in Cystic Fibrosis.

Manifestation	Key Findings	Prevalence	Detection Method
RV Dysfunction & Cor Pulmonale	Impaired RV strain (−22.0% vs. −29.5%; *p* < 0.001); mPAP rises from 27.8 to 47.2 mmHg on exercise	PH in 36–60% of transplant candidates	Strain echo, RHC, CT (PA:A ratio)
LV & Biventricular Dysfunction	Reduced LV strain despite preserved LVEF; LV hypertrophy driven by extracellular matrix expansion (ECV 27.6% vs. 24.8%; *p* = 0.030); diastolic dysfunction specific to CF vs. non-CF bronchiectasis	44% with abnormal cardiac function—single cross-sectional cohort (Duus et al., n = 104), composite of LV diastolic, LV systolic, and RV systolic abnormalities; not an independent second finding	Strain echo, TDI, cardiac MRI (T1/T2 mapping, ECV)
Arrhythmias & Electrical Remodeling	Dysregulated CFTR dosage arrhythmogenic in both knockout and overexpression models; borderline/prolonged QTc in ~20% on azithromycin	VAs in 6.7% on exercise testing; cardiac admissions increasing 16.4%/year	Exercise ECG, Holter, QTc monitoring
Atrial Remodeling	Biatrial dilation with impaired LA reservoir and conduit function	Documented in cohort study (n = 82)	Atrial strain echo
Tricuspid Valve Disease	Functional TR from RV pressure overload and annular dilation	TR jet in 67% of transplant candidates	Doppler echo
Pericardial Disease	Multifocal calcification (58%); progressive in 21%; associated with *B. cenocepacia* ET12	18% on chest CT (vs. 1% controls; single-center; replication pending)	Chest CT
Coronary Artery Disease	Two preclinical phenotypes: arterial stiffness (PI) vs. increased carotid IMT (PS); dyslipidemia worsens on ETI	CAD prevalence in CF not established; overweight/obesity rising in HEMT era (contextual only)	Carotid IMT, PWV, lipid panel
Heart Failure	Pre-HEMT: cor pulmonale; Post-HEMT: persistent biventricular dysfunction, emerging HFpEF phenotype	HF > 10× more prevalent in adult vs. pediatric CF admissions	Strain echo, cardiac MRI

This table summarizes the key cardiovascular manifestations identified in individuals with cystic fibrosis, including representative findings, estimated prevalence where available, and recommended detection modalities. Findings are drawn from both pre-HEMT and post-HEMT era studies. Abbreviations: CF, cystic fibrosis; CT, computed tomography; ECV, extracellular volume; ETI, elexacaftor/tezacaftor/ivacaftor; echo, echocardiography; ECG, electrocardiography; HEMT, highly effective modulator therapy; HF, heart failure; HFpEF, heart failure with preserved ejection fraction; IMT, intima-media thickness; LA, left atrium; LV, left ventricle; LVEF, left ventricular ejection fraction; mPAP, mean pulmonary artery pressure; MRI, magnetic resonance imaging; PA:A, pulmonary artery-to-aorta ratio; PH, pulmonary hypertension; PI, pancreatic insufficient; PS, pancreatic sufficient; PWV, pulse wave velocity; RHC, right heart catheterization; RV, right ventricle; TDI, tissue Doppler imaging; TR, tricuspid regurgitation; VAs, ventricular arrhythmias.

## Data Availability

No new data were created or analyzed in this study. Data sharing is not applicable to this article.

## Supplementary Materials

The following supporting information can be downloaded at: https://www.mdpi.com/article/10.3390/jcm15207988/s1. Supplementary Table S1: Design and sample size of pivotal evidentiary studies discussed in this review. Several key mechanistic and imaging findings derive from small, single-center studies (n as low as 8–10); these should be interpreted with caution given limited statistical power and the potential for publication bias favoring positive findings. Multinational and population-based cohorts (Frost, Arya) provide the strongest epidemiologic evidence.
