# Supplementary material for: Cardiovascular Manifestations of Cystic Fibrosis
Source: J Clin Med. 2026 Oct 15;15(20):7988. doi: 10.3390/jcm15207988 (PMC13705640; doi:10.3390/jcm15207988)
Supplement: Supplementary file 1 [file jcm-15-07988-s001.zip › jcm-4535805-supplementary.pdf]

Supplementary Material:

Study	Design	n	Center(s)	Key finding
Frost et al.[1]	Retrospective propensity-matched cohort	>11,000 CF	Multinational (multicenter)	65% higher MACE risk (HR 1.65)
Arya et al.[2]	Population-based cohort	Population-level	Canada (multicenter)	CVD at 2.9× rate, ≥20 y earlier (RR 2.9)
Duus et al.[3]	Cross-sectional, 1:1 matched	104 CF	Single-center (Denmark)	44% abnormal cardiac function (93% on modulators)
Rodriguez-Takeuchi et al.[4]	Cross-sectional CT	348 CF	Single-center	18% pericardial calcification
Dordevic et al.[5]	Cross-sectional strain echo	82 CF / 32 controls	Single-center	Biatrial remodeling
Lagan et al.[6]	Case-control CMR (pilot)	10 CF / 12 controls	Single-center	↑ ECV / extracellular matrix expansion
Sellers et al.[7]	Cross-sectional strain-rate echo (pilot)	8 CF adults (vs. literature values)	Single-center	Subclinical LV dysfunction (100%)
Eising et al.[8]	Cross-sectional, pediatric (3–12 y)	33 CF / 33 controls	Single-center	↓ LV/RV strain, ↑ PWV
Poore et al.[9]	Cross-sectional case-control	15 CF / 15 controls	Single-center	↓ brachial FMD

Zebrak et al.[10]	Autopsy/case series	18 infants	Multicenter registry	Infantile myocardial fibrosis/necrosis, SCD

Supplementary Table S1. Design and sample size of pivotal evidentiary studies discussed in this review. Several key mechanistic and imaging findings derive from small, single-center studies (n as low as 8–10); these should be interpreted with caution given limited statistical power and the potential for publication bias favoring positive findings. Multinational and population-based cohorts (Frost, Arya) provide the strongest epidemiologic evidence.

References:

1. Frost F, Nazareth D, Fauchier L, et al. Prevalence, risk factors and outcomes of cardiac disease in cystic fibrosis: a multinational retrospective cohort study. *Eur Respir J*. 2023;62(4):2300174. doi:10.1183/13993003.00174-2023

2. Arya R, Sharpe I, Cheng SY, et al. Elevated Rates and Earlier Onset of Nonpulmonary Comorbidities in Adults with Cystic Fibrosis: A Population-based Study. *Ann Am Thorac Soc*. 2025;22(12):1874-1880. doi:10.1513/AnnalsATS.202502-170OC

3. Duus LS, Dons M, Thudium RF, et al. Cardiac Structure and Function in People with Cystic Fibrosis. *J Cyst Fibros Off J Eur Cyst Fibros Soc*. 2024;23(6):1138-1145. doi:10.1016/j.jcf.2024.09.012

4. Rodriguez-Takeuchi S, Tullis E, Babaei Jandaghi A, et al. Association between Cystic Fibrosis and Pericardial Calcification Detected at Chest CT in Adults. *Radiology*. 2025;314(2):e241793. doi:10.1148/radiol.241793

5. Dordevic A, Genger M, Schwarz C, et al. Biatrial Remodeling in Patients with Cystic Fibrosis Running Title: Atrial Function in Cystic Fibrosis. *J Clin Med*. 2019;8(8):1141. doi:10.3390/jcm8081141

6. Lagan J, Naish JH, Bradley J, et al. Cardiac involvement in cystic fibrosis evaluated using cardiopulmonary magnetic resonance. *Int J Cardiovasc Imaging*. 2022;38(5):1121-1131. doi:10.1007/s10554-021-02496-6

7. Sellers ZM, McGlocklin L, Brasch A. Strain rate echocardiography uncovers subclinical left ventricular dysfunction in cystic fibrosis. *J Cyst Fibros Off J Eur Cyst Fibros Soc*. 2015;14(5):654-660. doi:10.1016/j.jcf.2015.03.010

8. Eising JB, van der Ent CK, Teske AJ, Vanderschuren MM, Uiterwaal CSPM, Meijboom FJ. Young patients with cystic fibrosis demonstrate subtle alterations of the cardiovascular system. *J Cyst Fibros Off J Eur Cyst Fibros Soc*. 2018;17(5):643-649. doi:10.1016/j.jcf.2017.12.009

9. Poore S, Berry B, Eidson D, McKie KT, Harris RA. Evidence of vascular endothelial dysfunction in young patients with cystic fibrosis. *Chest*. 2013;143(4):939-945. doi:10.1378/chest.12-1934

10. Zebrak J, Skuza B, Pogorzelski A, et al. Partial CFTR genotyping and characterisation of cystic fibrosis patients with myocardial fibrosis and necrosis. *Clin Genet*. 2000;57(1):56-60. doi:10.1034/j.1399-0004.2000.570108.x
